# Lactate/Pyruvate Ratio as an Early Predictor of Mortality in Patients with Sepsis: A Cohort Study

**DOI:** 10.3390/jcm13185597

**Published:** 2024-09-21

**Authors:** Olga G. Cantu-Rodriguez, Jose A. Hawing-Zarate, Edgar G. Dorsey-Trevino, David Hernandez-Barajas, Leonel E. Villalobos-Gutierrez, Jose Carlos Jaime-Perez, Consuelo Mancias-Guerra, Oscar Gonzalez-Llano, Graciela A. Gonzalez-Cantu, David Gomez-Almaguer, Cesar H. Gutierrez-Aguirre

**Affiliations:** 1 Department of Hematology, Hospital Universitario Dr. José Eleuterio González, Universidad Autonoma de Nuevo Leon, Monterrey 64460, Mexico; ogcantur@yahoo.com.mx (O.G.C.-R.); drhawing@gmail.com (J.A.H.-Z.); edgar.dorseytrevino@utrgv.edu (E.G.D.-T.); leon_vgtz@hotmail.com (L.E.V.-G.); carjaime@hotmail.com (J.C.J.-P.); consuelomanciasg@gmail.com (C.M.-G.); droscargonzalezllano@gmail.com (O.G.-L.); graciela.gonzalezcnt@gmail.com (G.A.G.-C.); dgomezalmaguer@gmail.com (D.G.-A.); 2 Department of Medicine, School of Medicine, The University of Texas Rio Grande Valley, Edinburg, TX 78539, USA; 3 Department of Internal Medicine, The University of Texas Rio Grande Valley, Weslaco, TX 78596, USA; 4 Department of Oncology, Hospital Universitario Dr. José Eleuterio González, Universidad Autonoma de Nuevo Leon, Monterrey 64460, Mexico; davidhdzb@hotmail.com

**Keywords:** sepsis, lactate, lactate/pyruvate ratio, pyruvate, mortality, prognosis, biomarker, systemic infection

## Abstract

**Background**: The lactate/pyruvate (LP) ratio has been studied as an alternative to serum lactate to determine clinical prognosis. Despite its clinical utility, there is a paucity of evidence evaluating the role of the L/P ratio in patients with sepsis. **Methods**: We assessed the clinical utility of the L/P ratio in patients with sepsis. The L/P ratio was measured at baseline, 4 and 8 h after admission. Our primary outcome was to determine the prognostic utility of the L/P ratio on the 15-day mortality risk. Our secondary outcomes were to compare the L/P ratio across time and its prognostic utility against standard risk calculators such as APACHE-II and SOFA scores. **Results**: We had a total of 80 patients, with 18 (22.5%) survivors and 62 (77.5%) non-survivors. While we found that patients having higher L/P ratios at 8 h had an increased 30-mortality risk (OR 1.08, 95% CI 1.02–1.18), the model’s performance showed no difference when compared to other measurements of the L/P ratio that showed no association with mortality (*p*-value: 0.45). For our secondary outcome, we found that the APACHE-II and SOFA scores have better performance and predictability than the L/P ratio (AUC 0.83 and AUC 0.80, respectively), but showed no association with mortality (OR 1.07, 95% CI 1.01–1.17 and OR 1.08, 95% CI 1.02–1.18). **Conclusions**: Based on our findings, the L/P ratio appears to function more effectively as an early predictor of mortality when used as an adjuvant biomarker with other clinical parameters.

## 1. Introduction

The clinical prognosis for patients with sepsis remains challenging due to the lack of reliable biomarkers that would aid clinicians in determining severity and guiding prognosis [1]. Serum lactate is commonly measured as part of the 1 h bundle laboratory testing process, as it is closely related to tissue hypoxia and mortality [2,3]. Nevertheless, non-hypoxic factors such as decreased lactate clearance [4], inhibition of pyruvate dehydrogenase [5], or accelerated glycolysis [6,7] could increase serum lactate without a factual tissue hypoperfusion state [8], potentially deluding clinicians when evaluating disease severity, leading to misguided treatment decisions and misjudged prognoses.

An alternative to control for serum lactate’s inherent variability is to measure serum pyruvate concomitantly to calculate the lactate/pyruvate (L/P) ratio [9,10]. Pyruvate, an intermediate substrate in anaerobic lactate production, decreases serum levels during tissue hypoperfusion as it is converted to lactate, thereby increasing the L/P ratio [11,12]. Studies evaluating the clinical utility of the L/P ratio as a prognostic biomarker have consistently shown that elevation in the L/P ratio was associated with an increase in mortality risk [9,13,14,15], whereas high serum lactate levels but a lack of increase in the L/P ratio had no association with mortality risk [16]. While previous studies have established the association between the L/P ratio and mortality risk in critically ill patients or with an established circulatory shock [17,18,19], to the best of our knowledge, there is a paucity of studies evaluating the role of the L/P ratio as a prognostic marker in patients with sepsis without evidence of shock.

To explore the potential clinical utility of serum pyruvate as a prognostic biomarker, we conducted a prospective cohort study to evaluate the L/P ratio’s prognostic significance in patients with sepsis. We hypothesized that patients with sepsis with higher L/P ratios would have an increased 15-day mortality risk.

## 2. Material and Methods

### 2.1. Patient Selection and Study Design

This prospective cohort study was conducted to evaluate clinical outcomes in patients who were admitted due to sepsis. This study followed institutional guidelines and received approval from the Institutional Review Board and the Ethics Committee of the School of Medicine. It also follows the Strengthening the Reporting of Observational Studies in Epidemiology (STROBE) guidelines [20].

Patients arriving at the emergency department with clinical signs of sepsis received routine clinical care based on their cardiorespiratory needs, which included supportive therapy and empiric antimicrobial therapy. A venous blood gas analysis was taken to measure lactate and pyruvate at baseline, 4, and 8 h after admission. Baseline measurements were taken at the emergency department, whereas measurements at 4 and 8 h depended on the patient’s location. To evaluate the disease severity in each patient, we measured the Acute Physiologic and Chronic Health Disease Classification System II (APACHE II) [21] and the Sequential Organ Failure Assessment (SOFA) at baseline [22].

Eligible patients were at least 18 years old, with a diagnosis of sepsis regardless of etiology. Sepsis was defined as life-threatening organ failure caused by the host’s inappropriate response to infection, as determined by the Surviving Sepsis Campaign criterion [23]. A patient was considered to be in sepsis if they presented with a potential source of infection plus two of the four features used for systemic inflammatory response syndrome (SIRS): white blood cells < 4000/mm^3^ or >10,000/mm^3^; heart rate > 90 bpm; respiratory rate > 20/min or a PaCO_2_ < 32 mmHg; or body temperature < 36 °C or >38 °C. Patients with a history of underlying mitochondrial and/or enzymatic diseases; who reported alcohol consumption in the last 24 h; had clinical evidence of septic shock based on the use of vasopressors on admission; were pregnant; had a medical background of chronic liver disease, chronic kidney disease, chronic obstructive pulmonary disease, asthma, oncological or hematological malignancy, or rheumatological diseases; or who self-reported or were found to have a polysubstance abuse disorder were excluded. Lastly, patients with an initial clinical presentation suggestive of infectious sepsis that was later ruled out were eliminated from the study. The Institution’s ethics committee approved the study, which was conducted based on good clinical practices.

### 2.2. Laboratory Measurements

Serum lactate was measured by venous blood gas analysis using a GEM 5000 premier (Werfen, Barcelona, Spain). A Cayman Chemical Item No. 700470 Assay Kit was used for serum pyruvate measurement. Samples were drawn in a vacutainer tube without anticoagulant, waiting 30 min for clot formation at 25 °C. Subsequently, the samples were centrifuged at 2000× *g* for 15 min at 25 °C, and the supernatant was extracted without contacting the layer of leukocytes. For every 500 µL of serum, 500 µL of MPA was added, vortexed, and placed on ice for 5 min. The samples were centrifuged again at 10,000× *g* for 5 min at 4 °C. The resulting supernatant was removed before adding 50 µL of potassium carbonate. Centrifugation was again carried out at 10,000× *g* for 5 min, and the supernatant was frozen at −80 °C. Serum samples were frozen and processed together (after thawing) a second time. The samples were preserved at −80 °C for an average of 42 days (2–76) and were analyzed simultaneously after thawing.

### 2.3. Outcomes

Our primary outcome was to determine the prognostic utility of the L/P ratio on 15-day mortality risk. Our secondary outcomes were to compare the L/P ratio across time and its prognostic utility against the APACHE-II or SOFA scores. Primary and secondary outcomes were assessed up to 15 days of hospitalization or mortality, whichever came first.

### 2.4. Statistical Analysis

For the primary outcome, an additive regression model for the L/P ratio at baseline, 4 h, and 8 h was performed to predict 15-day mortality risk. Similarly, for our secondary outcomes, additive regression models for the APACHE-II, SOFA score, and the L/P ratio measured at 8 h were performed to predict 15-day mortality risk. After we obtained the model’s performance, we selected the model with the higher predictability performance and compared it against the APACHE-II and SOFA scores. Model performance was evaluated based on the residual mean square error (RMSE), R^2^, C-statistic, accuracy, and a visual inspection of the receiver operating characteristic (ROC) curve. Model comparison was made with ANOVA and considered a *p*-value < 0.05 as statistically significant. Furthermore, a non-parametric pairwise comparison was made to determine differences between survivors and non-survivors on the L/P ratio across time.

All models included the following covariates: comorbidities, age, sex, source of infection, empiric antibiotic use, comorbidities, and length of stay at the intensive care unit. Additionally, we included high-order polynomials for all numerical variables in the model to ensure the model’s flexibility, and we added an interaction term between the source of infection and length of stay as we believe these two variables provide more information when considered together. A non-parametric bootstrap regression was used for all models, given that we expect a non-linear relationship between variables to allow for more model flexibility. Resampling was conducted using 1000 iterations to calculate coefficients and percentile-based 95% confidence intervals (CI). The bootstrap populations were resampled from the entire population.

We conducted two sub-analyses and compared the L/P ratio across time between survivors and non-survivors. For the first sub-analysis, we divided patients based on their source of infection. For the second sub-analysis, we divided patients based on the severity of the L/P ratio, APACHE-II, and SOFA scores. Patients were considered to have severe initial clinical presentation if they had an L/P value of ≥2.5 at 8 h, an APACHE-II of ≥20, or a SOFA of ≥10. The three-way comparison was made using the non-parametric Kruskal–Wallis test with Bonferroni correction for multiple comparisons, whereas a pairwise comparison was created using the Wilcoxon test.

All the statistical analyses were performed using R statistical software (version 4.2.3, R core Team 2023).

## 3. Results

A total of 80 participants were included in the analysis that had a median age of 48 (IQR 38–58), SOFA of 7.5 (IQR 5–10), APACHE-II of 15 (IQR 10–24), and an L/P ratio of 1.2 (0.58–1.70) (Table 1). Overall, pneumonia was the most common source of infection (44/80; 55%), and gastrointestinal was the least common (15/80; 19%) (Table 1). There were no differences between survivors and non-survivors other than the APACHE-II score and SOFA scores (27, IQR 22–36 vs. 12, IQR 9–20, *p*-value ≤ 0.001; 11, IQR 9.25–12.75, *p*-value ≤ 0.001, respectively) (Table 1).

**Figure 1:**
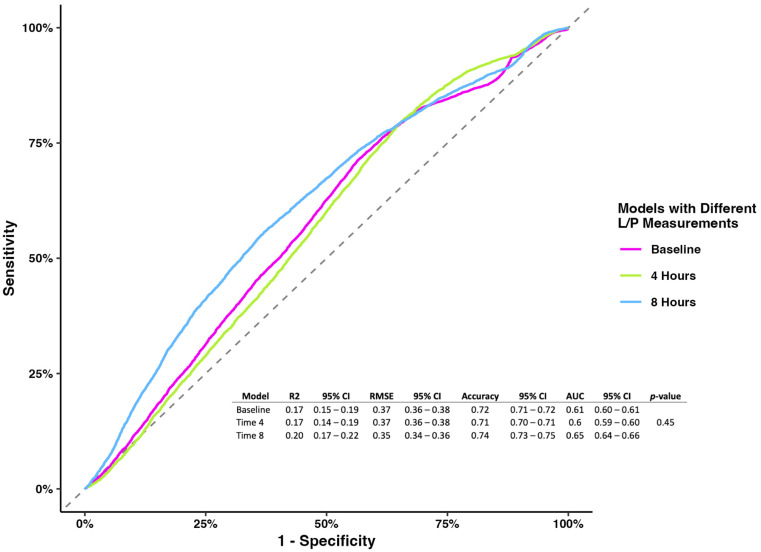
Receiver operating characterization and metrics for models of the L/P ratio at baseline, 4 h, and 8 h.

The prognostic model evaluating the L/P ratio measured at different time points showed no association at baseline and 4 h. Still, the L/P ratio measured at 8 h showed that higher levels were associated with mortality (OR 1.08, 95% CI 1.02–1.18), holding the rest of the variables constant (Table 2). While the models’ metrics showed that the L/P ratio measured at 8 h had better performance than the other two models (accuracy 0.74, 95% CI 0.73–0.75; AUC 0.65, 95% CI 0.64–0.66), the model comparison showed no difference (*p*-value = 0.45) (Figure 1).

For the APACHE-II and SOFA scores, we found no association with mortality (OR 0.97, 95% CI 0.96–1.12 and OR 0.94, 95% CI 0.91–1.09, respectively) (Table 3).

**Figure 2:**
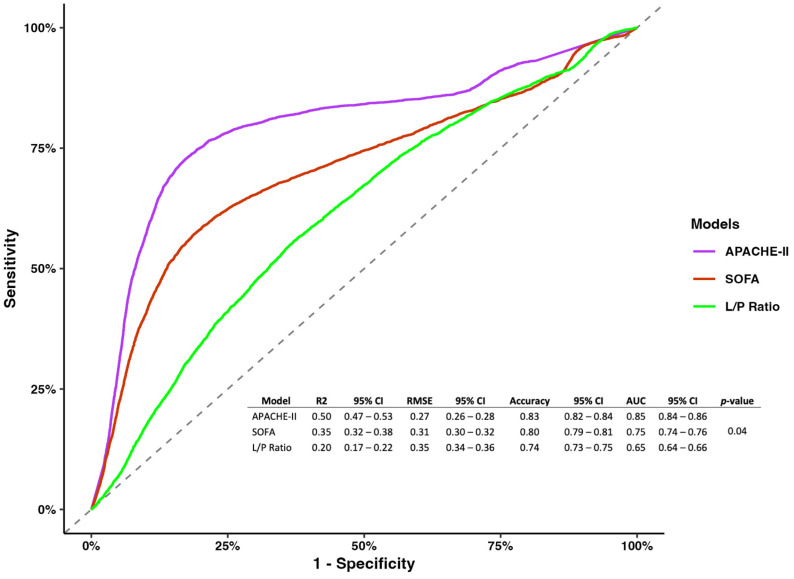
Receiver operating characterization for models of the APACHE-II, SOGA, and L/P ratio measured at 8 h.

However, regarding model metrics, the APACHE-II showed better performance and predictability than the SOFA, and the L/P ratio measured at 8 h (R2 0.5, 95% CI 0.47–0.53; RMSE 0.27, 95% CI 0.26–0.28; accuracy 0.83, 95% CI 0.82–0.84; AUC 0.85 0.84–0.86; *p*-value = 0.04) (Figure 2).

**Figure 3:**
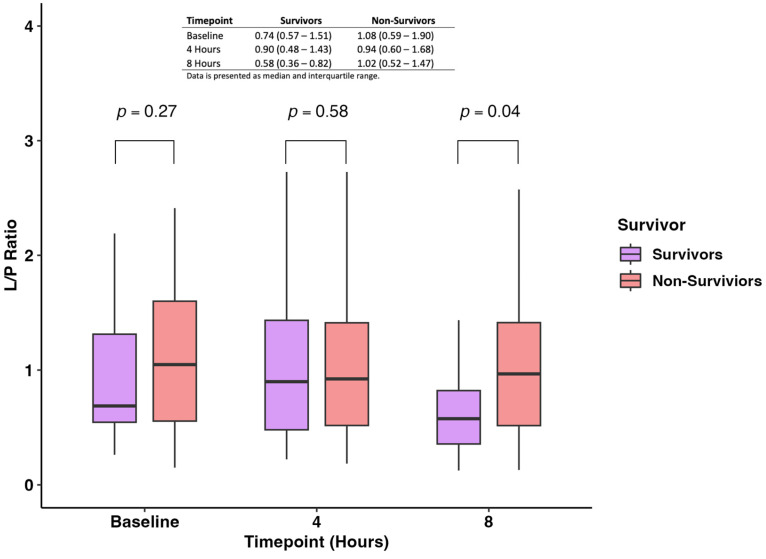
Levels of the L/P ratio were measured at baseline, 4 h, and 8 h between survivors and non-survivors.

The pairwise comparison of the L/P ratio measured at baseline, 4 h, and 8 h showed no difference between survivors and non-survivors at baseline and 4 h (*p*-value: 0.27 and 0.58, respectively) (Figure 3). On the other hand, we found that non-survivors had statistically significantly higher levels of L/P ratio when compared with survivors (0.58; IQR 0.36–0.82 vs. 1.02; IQR 0.52–1.47; *p*-value = 0.04) (Figure 3).

**Figure 4:**
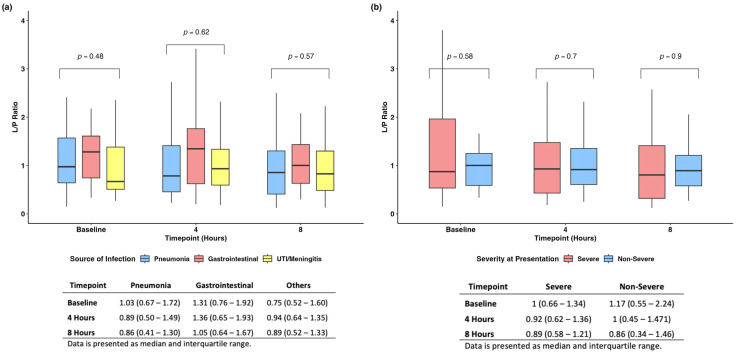
Subanalyses based on source of infection (**a**) and severity of disease at presentation (**b**).

We found no difference when evaluating the L/P ratio among the different sources of infections and at different time points (Figure 4a). Similarly, when stratifying patients based on disease severity at baseline, we found no difference in the L/P ratio across time (Figure 4b).

## 4. Discussion

The measurement of the L/P ratio has emerged as a promising clinical biomarker for the treatment and prognosis of critically ill patients, particularly those experiencing shock. However, its clinical utility in sepsis patients without shock has not been thoroughly evaluated. In our prospective observational study, we aimed to investigate the association between the L/P ratio and 15-day mortality in patients with severe sepsis. Our findings revealed that persistently elevated L/P ratios were associated with an increased mortality risk. Nonetheless, its prognostic performance for predicting 15-day mortality was suboptimal compared to established scoring systems such as APACHE-II and SOFA. Based on these findings, the L/P ratio’s role as an early predictor of mortality in sepsis appears promising as an adjuvant test with other clinical scores to provide a more robust evaluation.

Our findings of higher levels of the L/P ratio being associated with higher 15-day mortality risk resonates with previous evidence. For instance, the observational study by Nikitas et al. found that higher L/P ratios in adipose tissue were associated with an increase in 28-day mortality risk in patients with septic shock [24]. Similarly, Suistoma et al. performed a prospective cohort study where they included patients in the emergency department, finding that elevations in the L/P ratio were associated with tissue hypoperfusion and an increased mortality risk [18]. Similar findings were seen by Levy and colleagues, who sought to measure the L/P ratio in patients with septic shock that required catecholamine therapy, finding that persistently elevated levels of the L/P ratio were associated with multiorgan failure [15]. Furthermore, we found that the prognostic performance of the L/P ratio measured at 8 h was suboptimal compared to the APACHE-II and SOFA scores. This, however, was an expected finding, as the APACHE-II and the SOFA scores consider a wide array of clinical and laboratory variables that allow for a more robust approach to patient care.

Before integrating the L/P ratio into routine patient care, several research avenues must be explored and consolidated. Firstly, our findings suggest that the L/P ratio is more effective at differentiating true tissue hypoperfusion from other causes of hyperlactatemia. Future studies should further investigate this association through extended evaluations of the L/P ratio in patients with sepsis and other conditions. Secondly, our study excluded patients with chronic kidney disease due to potential impacts on serum levels. Future research should assess the utility of the L/P ratio in this population. Lastly, our findings could be extended to surgical patients who underwent operations due to infectious causes or developed post-operative sepsis, offering further insights into the L/P ratio’s clinical applicability.

While our study provides evidence for the clinical utility of the L/P ratio in patients with sepsis without shock, several limitations must be acknowledged. Firstly, the majority of patients had pneumonia as their infection source, which could have skewed our results. However, this scenario also demonstrates the L/P ratio’s applicability in varied and complex clinical contexts, a premise further supported by our subanalysis showing no significant differences in the L/P ratio across different infection sources. Secondly, unlike previous studies that measured the L/P ratio over several days, we were limited to measurements taken on the day of admission. Nonetheless, our findings, which align with studies involving prolonged measurements, suggest that daily monitoring may be unnecessary. Fourth, our study lacks information regarding the type of bacterial infection, which could bias our results as Gram-negative bacteria produce more lactic acid than Gram-positive bacteria [25,26,27]. Future research could evaluate if the L/P ratio is affected based on the type of bacteria. Fifth, our study was conducted in a single center, which could influence our results, as each location has its own resources, limitations, and protocols. Sixth, given that our definition of sepsis was merely based on objective clinical parameters due to the unavailability of sophisticated tools to aid in classification, our results might be biased due to the possibility of suboptimal sensitivity and specificity when classifying patients. Nevertheless, classifying patients based on clinical parameters remains the standard of care in many clinical settings where sophisticated tools, such as machine learning models, are widely unavailable. We, however, acknowledge that future research could leverage our results to determine if using sophisticated classification tools would find significantly different results than ours. Lastly, our small sample size prevented us from validating our prognostic model. Despite this, our results are consistent with existing evidence, and the robustness of our model indicates that a more extensive, validated study would likely yield similar outcomes.

## 5. Conclusions

Our study addresses existing knowledge gaps regarding the clinical utility of the L/P ratio in critically ill patients, particularly in distinguishing serum lactate elevations due to factors other than hypoperfusion. Through an observational study, we provided evidence supporting the application of the L/P ratio in patients with sepsis. Our findings indicate that persistently elevated L/P ratios are associated with an increased risk of 15-day mortality, but its prognostic performance was suboptimal compared to conventional risk scores. Thus, the L/P ratio appears to function more effectively as an early predictor of mortality when used as an adjuvant biomarker with other clinical parameters.

## Figures and Tables

**Table 1:** Demographic characteristic of participants at baseline.

Characteristic	Overall n = 80	Survivors (n = 18)	Non-Survivors (n = 62)	*p*-Value
Age	48 (38–58)	44.67 (16.82)	48.82 (16.90)	0.4
Sex				
Female	47 (59)	12 (66.6)	35 (56)	0.4
Charlson Comorbidity Index	2.94 (1.64	3 (1.61)	3.06 (1.63)	0.8
SOFA	7.5 (5–10)	11 (9.25–12.75)	7 (5–9)	<0.001 *
APACHE-II	15 (10–24)	27 (22–36)	12 (9–20)	<0.001 *
Lactate (mg/dL)	1.90 (1.1–4)	1.10 (1.07–3.63)	2 (1.30–3.95)	0.08
Pyruvate (mg/dL)	1.8 (1.5–2.8)	1.65 (1.53–2.65)	1.90 (1.50–2.78)	0.6
L/P Ratio	1.2 (0.58–1.70)	0.74 (0.57–1.51)	1.08 (0.59–1.90)	0.3
Source of Infection				0.1
Pneumonia	44 (55)	13 (72)	31 (50)	
Urinary tract infection	19 (23.75)	1 (5.6)	18 (29)	
Gastrointestinal	15 (19)	1 (5.6)	20 (32)	
Meningitis	2 (2.5)	0 (0)	2 (3.2)	
Comorbidities				0.7
Congestive Heart Failure	44 (55)	8 (44.4)	34 (54.8)	
History of Myocardial Infarction	16 (20)	3 (16.7)	12 (19.4)	
Peripheral Arterial disease	46 (57.5)	11 (61.1)	33 (53.2)	
Type 2 Diabetes Mellitus	25 (31)	7 (39)	18 (29)	
Hypertension	38 (48)	7 (39)	31 (50)	

Data are presented as median and interquartile ranges and proportions with percentages unless specified otherwise. Others source of infection included urinary tract infection and meningitis. SOFA: Sequential Organ Failure Assessment; APACHE: Acute Physiology and Chronic Health Evaluation; L/P Ratio: Lactate/Pyruvate Ratio. * Statistically significant.

**Table 2:** Estimates to predict mortality based on the L/P ratio measured at different timepoints.

Predictor	Baseline		4 h		8 h	
	OR	95% CI	OR	95% CI	OR	95% CI
L/P Ratio	1.02	0.96–1.08	1.03	0.97–1.08	1.08	1.02–1.18 *

* Statistically significant. L/P Ratio: Lactate/Pyruvate Ratio; OR: Odds Ratio; CI: Confidence Interval.

**Table 3:** Estimates of different models to predict mortality using different risk calculators and the L/P ratio.

Predictor	APACHE-II		SOFA		L/P Ratio	
	OR	95% CI	OR	95% CI	OR	95% CI
APACHE-II	0.97	0.96–1.12	-	-	-	-
SOFA	-	-	0.94	0.91–1.09	-	-
L/P Ratio	-	-	-	-	1.08	1.02–1.18 *

* Statistically significant. Other sources of infection included urinary tract infection and meningitis. L/P Ratio: Lactate/Pyruvate Ratio; OR: Odds Ratio; CI: Confidence Interval.

## Data Availability

All the data generated or analyzed during this study are included in this published article.
